# A Micelle Self-Assembled from Doxorubicin-Arabinoxylan Conjugates with pH-Cleavable Bond for Synergistic Antitumor Therapy

**DOI:** 10.1186/s11671-017-1836-z

**Published:** 2017-01-25

**Authors:** Jie Wang, Yanli Li, Xia Dong, Ying Wang, Xiaodan Chong, Tai Yu, Fulei Zhang, Di Chen, Li Zhang, Jie Gao, Cheng Yang, Jun Han, Wei Li

**Affiliations:** 1 College of Pharmacy & Institute of Biopharmaceutical Research, Liaocheng University, 1 Hunan Road, Liaocheng, Shandong 252000 People’s Republic of China; 2 International Joint Cancer Institute, The Second Military Medical University, 800 Xiangyin Road, Shanghai, 200433 People’s Republic of China; 3 School of Chemical and Material Engineering, Jiangnan University, 1800 Lihu Avenue, Wuxi, 214122 People’s Republic of China

**Keywords:** Biocompatibility, Micelle, pH-cleavable bond, Nanomedicine, Synergistic antitumor therapy

## Abstract

**Abstract:**

Nanomedicine offers new hope to overcome the low solubility and high side toxicity to normal tissue appeared in traditional chemotherapy. The biocompatibility and intracellular drug accumulation is still a big challenge for the nano-based formulations. Herein, a medical-used biocompatible arabinoxylan (AX) is used to develop to delivery chemodrug doxorubicin (DOX). The solubility of DOX is obviously enhanced via the hydrogen bond formed with AX which results in an amphiphilic AX-DOX. A micelle with pH-cleavable bond is thus self-assembled from such AX-DOX with DOX core and AX shell. The inner DOX can be easily released out at low intracellular pH, which obviously enhanced its in vitro cytotoxicity against breast cancer cells (MCF-7). Interestingly, an unexpected apoptosis is evoked except for the proliferation inhibition. Moreover, the therapeutic effects are further synergistically promoted by the enhanced permeability and retention (EPR) and intracellular pH-triggered drug release. Consequently, the in vivo intratumor accumulation of DOX, the tumor inhibition was significantly promoted after intravenous administration to the Balb/c nude mice bearing MCF-7 tumors. These in vitro/vivo results indicated that the AX-DOX micellular formulation holds high potential in cancer therapy.

**Graphical Abstract:**

## Background

The small molecular chemodrug DOX is widely used in clinic due to its high cytotoxicity against many tumors, including liver cancer, lymphoma, gastric cancer, and breast cancer [1–4]. The clinic merit is strongly limited by its side effects to the normal tissue which is attributed to its low aqueous solubility, quick degradation, and poor in vivo tumor-targeting capability [5]. Therefore, development of new DOX formulation is highly desired. Nanomedicine offers new hope in overcoming the abovementioned drawbacks [6, 7] by some advantages such as in vivo stability [8], controlled drug dosage [9], and low toxicity [10]. Many nano formulations based on liposomes [11], nano-gel [12], and polymeric micelles [13] have been extensively investigated recently. For further promoting their therapeutic index, the biocompatibility of the carriers and intracellular drug distribution are still needed to improve [6, 7, 14].

Noted, the AX is a natural polysaccharide and has been used in the medical field for the advantages of good biocompatibility and amphiphilicity [15] which is confirmed in its clinical trials and used in food [16, 17]. Moreover, AX is a chemosensitizing agent in the treatment of cancer [18]. Additionally, there are many –NH_2_ and –OH groups along the AX chain backbone, which deems the AX should be an excellent candidate for design of novel pH-sensitive nanomedicine with acid-responsive hydrogen bond [19]. In such case, the solubility of DOX can be enhanced via the hydrogen bond and hydrophobic interaction. On the other hand, the stability of AX-based nanomedicine can be promoted by the hydrophilic AX. The inner DOX can be easily released out at relatively low pH [20]. It is well known that the pH value of the normal cells is ~7.3. However, the pH in tumor is around 6.2–6.9. In some organelles such as endosomes and lysosomes, the acidity lowers to 4.5–6.0 [21]. This pH difference between tumor and non tumor cells offers an opportunity to control the intracellular drug release because the micelles can stably circulated in physiological conditions (pH 7.3) and release drug at the intracellular low pH.

In this study, a AX-DOX micelle was facilely prepared through hydrogen bond and hydrophobic interactions [19]. The biocompability of AX and the pH cleavable AX-DOX bonds were successfully utilized in this case. The nanoparticle’s properties, in vitro/vivo synergistic antitumor effects and corresponding mechanism, were systemically investigated. This study provided an easy and feasible idea for the design and preparation of pH-sensitive nano delivery system.

## Methods

### Materials

The AX was provided by the School of Chemical and Material Engineering, Jiangnan University. Doxorubicin hydrochloride (DOX∙HCl) was purchased from Dalian Meilun bio Co., Ltd. (Dalian, China). Methanol, acetone, chloroform, acetonitrile, chloroquine (CQ), and 3-methyladenine (3-MA) were bought from Sigma (St. Louis, MO, USA). *N*,*N*-dimethylacetamide (DMAC) and triethanolamine (TEA) were of analytical grade and were used without further purification. All organic reagents were of analytical grade and purchased from Sinopharm (Shanghai, China) unless specifically mentioned otherwise.

For in vitro cell culture, fetal bovine serum (FBS), the Dulbecco’s modified Eagle’s medium (DMEM) cell culture media, penicillin, and streptomycin were purchased from Invitrogen (Carlsbad, CA, USA). The cell counting kit-8 (CCK-8) was purchased from Dojindo laboratories (Kumamoto, Japan). An Annexin V-FITC/PI Apoptosis Detection Kit was purchased from Becton, Dickinson and Co. (NJ, USA).

Water was purified using a Milli-Q Synthesis A10 system (Millipore, Billerica, MA) in terms of resistivity 18.2 MΩ cm.

### Synthesis of Micelles

Typical synthesis procedure was outlined as follows: AX was dissolved in 1 mL DMAC (5 mg/mL). In order to fully dissolve, the mixture was stirred for 10 min at 120 °C with oil bath heating. The reaction system was cooled naturally to reach room temperature before adding the DOX mixture. DOX∙HCl was also dissolved in 1 mL DMAC (5 mg/mL). We prepared a TEA solution with 1.5 M equivalents to the DOX in DMAC and made tenfold dilution with DMAC. The TEA solution was added slowly dropwise into the DOX solution and kept stirring for about 5 min. Then, the DOX and the AX solution were mixed at a drug-to-nanocarrier feeding ratio of 1:1 and stirred for 24 h in dark at room temperature (RT). Afterward, the free DOX was removed by dialyzing against PBS using the dialysis membrane (molecular weight cutoff (MWCO), 1 kDa) in dark at RT, and this dialysis was kept for about 24 h with regularly replacing fresh PBS every 8 h. Finally, the AX-coated DOX (i.e., AX-DOX) micelles were collected and stored at 4 °C for further use.

In this article, the DOX is hydrophobic doxorubicin unless specifically mentioned otherwise.

### Characterization of micelles

The morphology of micelles was characterized using scanning electron microscopy (SEM) (Hitachi, Tokyo, Japan). The SEM experiments were conducted by depositing 10 μl of aqueous solutions of the micelles on a silicon chip and allowing them to dry for 60 min in air. Samples were imaged with an SEM. The conventional SEM images were obtained at 1.0 kV.

The hydrodynamic diameter and size distribution of micelles were studied by dynamic light scattering (DLS, ALV/CGS-3, Germany) instrument by dispersing the micelles (1 mg/mL) in Milli-Q water at the scattering angle of 90°.

### DOX Encapsulation Efficiency Studies

The encapsulation efficiency (EE) and the drug loading capacity (DLC) of DOX in the micelles were calculated by the following equations:

1 $$ \mathrm{E}\mathrm{E}={M}_{\mathrm{Encapsulated}}/{M}_{\mathrm{Fed}}\times 100\% $$

2 $$ \mathrm{D}\mathrm{L}\mathrm{C} = {M}_{\mathrm{Encapsulated}}/{M}_{\mathrm{Total}}\times 100\% $$

where *M*_Encapsulated_ represents the weight of DOX encapsulated in the micelles, *M*_Fed_ is the total weight of DOX fed for encapsulation, and M_Total_ is the total weight of micelles including both the encapsulated DOX and the non-DOX materials for making the empty micelles. The amount of DOX was dissolved in acetonitrile and vigorously vortexed to gain a solution at a drug-to-nanoparticle feeding ratio of 1:1 and then determined using UV-Vis spectrophotometer (Cary300, Varian, CA, USA) based on the absorbance at 485 nm.

### DOX Release Studies

The DOX release profiles were detected by a fluorescence spectrofluorometer (Cary Eclipse, Varian, CA, USA) using three different buffers: PBS 7.4, PBS 6.5, and acetate buffer at pH 4.5, which represent the pH of blood, the pH of the tumor microenvironment, and the pH of lysosomes and endosomes [22], respectively. Release studies from micelles were prepared as the following: 1 mL of micelles solution including the same concentration of DOX were transferred into dialysis bags (MWCO 3.5 kDa) that were placed in 100 mL of release buffer at 37 °C for 48 h. Periodically (0.5, 2, 4, 6, 8, 10, 12, 14, 16, 18, 20, 22, 24, 28, 32, 36, 40, 44, and 48 h), 0.5 mL of release medium was removed replaced with new buffer. DOX concentration and percentage of released DOX during a period of time were detected at 485 nm.

### Cell Lines and Culture

The human breast cancer MCF-7 cell line was purchased from American Type Culture Collection (Manassas, VA). The cell line was authenticated twice by morphologic and isoenzyme analyses during the study period. Cell lines were routinely checked for contamination by mycoplasma using Hoechst staining and consistently found to be negative. The cells were maintained at 37 °C in 5% CO_2_ in DMEM supplemented with 10% FBS, 100 U/mL penicillin, and 100 μg/mL streptomycin (Invitrogen, Carlsbad, CA). Medium was changed every other day.

### Cytotoxicity Assays

For cytotoxicity assays, the MCF-7 cells were seeded into 96-well plates (0.1 ml/well, 5 × 10^3^ cells/well) and incubated overnight until the cells reached 80% confluence. The cells were treated with fresh medium containing a known concentration of drug formulations ranging from 0.1 to 20 μg/mL in each well in triplicates, and the plates were incubated for 48 h. Another experiment, MCF-7 cells were co-treated with micelles at the concentration equivalent to 2 μg/mL DOX and 10 mM 3-MA or 60 μM CQ. The MCF-7 cells were incubated with those regents for 24 h.

Before harvest, the cytotoxicity was evaluated by adding 10 μL of CCK-8 solution to each well of the plate. After incubation for 1 h, the absorption of the samples in each well was measured using a BIO-TEK ELx800 Universal Microplate Reader (Bio-Tek, VT, USA) at wavelengths of 450 and 630 nm. The cell survival rate was calculated with the following formula: [(*A*_E_ − *A*_B_)/(*A*_C_ − *A*_B_)] × 100%, where *A*_E_, *A*_C_, and *A*_B_ represent the absorbance of the experimental cells [23], control cells, and background, respectively.

The IC_50_ of the drugs was calculated by the CompuSyn software (Chou and Martin, 2005, Compusyn, Inc., USA).

### Cellular Uptake Analyses

The cellular uptake behaviors of the nanoparticle in MCF-7 were analyzed using both flow cytometry (BD biosciences, CA) and confocal laser scanning microscopy (CLSM) (Carl Zeiss Meditec AG, Jena, Germany). For flow cytometry analyses, cells were seeded in 12-well plates at a density of 2 × 10^5^ cells/mL and incubated overnight. Then, the cells were treated with AX-DOX, DOX at a concentration of 2 μg/mL, and the culture medium was used as a blank control. After incubation for 8 h, the cells were washed with PBS. The cellular uptake of drugs was analyzed using a flow cytometer and FlowJo analysis software. For each sample, at least 2 × 10^4^ cells were analyzed.

For the CLSM studies, the MCF-7 cells were precultured in confocal laser scanning dishes at a density of 2 × 10^5^ cells per well overnight. The cells were treated with AX-DOX, DOX at a concentration of 2 μg/mL, and co-incubated with 10 mM 3-MA or 60 μM CQ for 8 h, respectively. The cells were then washed with PBS. Untreated cells were used as control. The cellular uptake was observed with a CLSM. Digital monochromatic images were acquired using ZEN Light Edition Software.

### Apoptotic Cells Evaluated by Flow Cytometry

The antitumor activity of different drug formulations was analyzed as described below. Briefly, MCF-7 cells were seeded into 24-well plates (5 × 10^4^ cells/well) and incubated overnight. After the cells reached 80% confluence, they were treated with drug at 37 °C with 5% CO_2_ for 48 h. The cells were trypsinized, collected, washed, and finally suspended in one-time binding buffer, followed by staining with an Annexin-V antibody labeled with Alexa Fluor-488 for 15 min at RT in the dark. Then, the apoptotic cells were analyzed by flow cytometry [24]. For each sample, at least 1 × 10^4^ cells were analyzed.

### In Vivo Anticancer Study

Female Balb/c nude mice (4 weeks, ~20 g) were obtained from the Shanghai Experimental Animal Center of Chinese Academic of Sciences (Shanghai, China) and kept under specific pathogen-free (SPF) conditions. They were allowed to acclimate 1 week in the animal facility to reduce stress after arrival, and all efforts were made to minimize animal suffering. A subcutaneous MCF-7 tumor xenograft mouse model was established by subcutaneously implanting 2 × 10^7^ MCF-7 cells into the right back of each 5-week-old female mouse. Two weeks after inoculation, mice with palpable tumors about 50 mm^3^ were randomized into three groups (5 mice per group). The three groups of tumor-bearing mice were injected with (1) PBS (control), (2) AX-DOX (5 mg/kg BW), (3) saturated aqueous solution of hydrophobic DOX (5 mg/kg BW) every 3 days for a total of three treatments. A total of 100 μL of PBS was used as the carrier for all drug formulations. Tumor volumes were measured by an external caliper every 3 days and were then calculated by the modified ellipsoidal formula: Tumor volume = (length × width^2^)/2. For the analysis of systemic toxicity of treatment, the body weight change was monitored at the same time. The mice were euthanized on day 33 after the drug injection. All animals were treated in accordance with guidelines of the Committee on Animals of the Second Military Medical University (Shanghai, China).

### Statistical Analysis

All data are reported as mean ± standard deviation (SD) from at least three independent runs. Statistical analysis of significance was calculated using Student’s *t* test. *p* < 0.05 is considered as statistically significant.

## Results and Discussion

### Synthesis and Characterization of Micelles and Drug Encapsulation

**Fig. 1:**
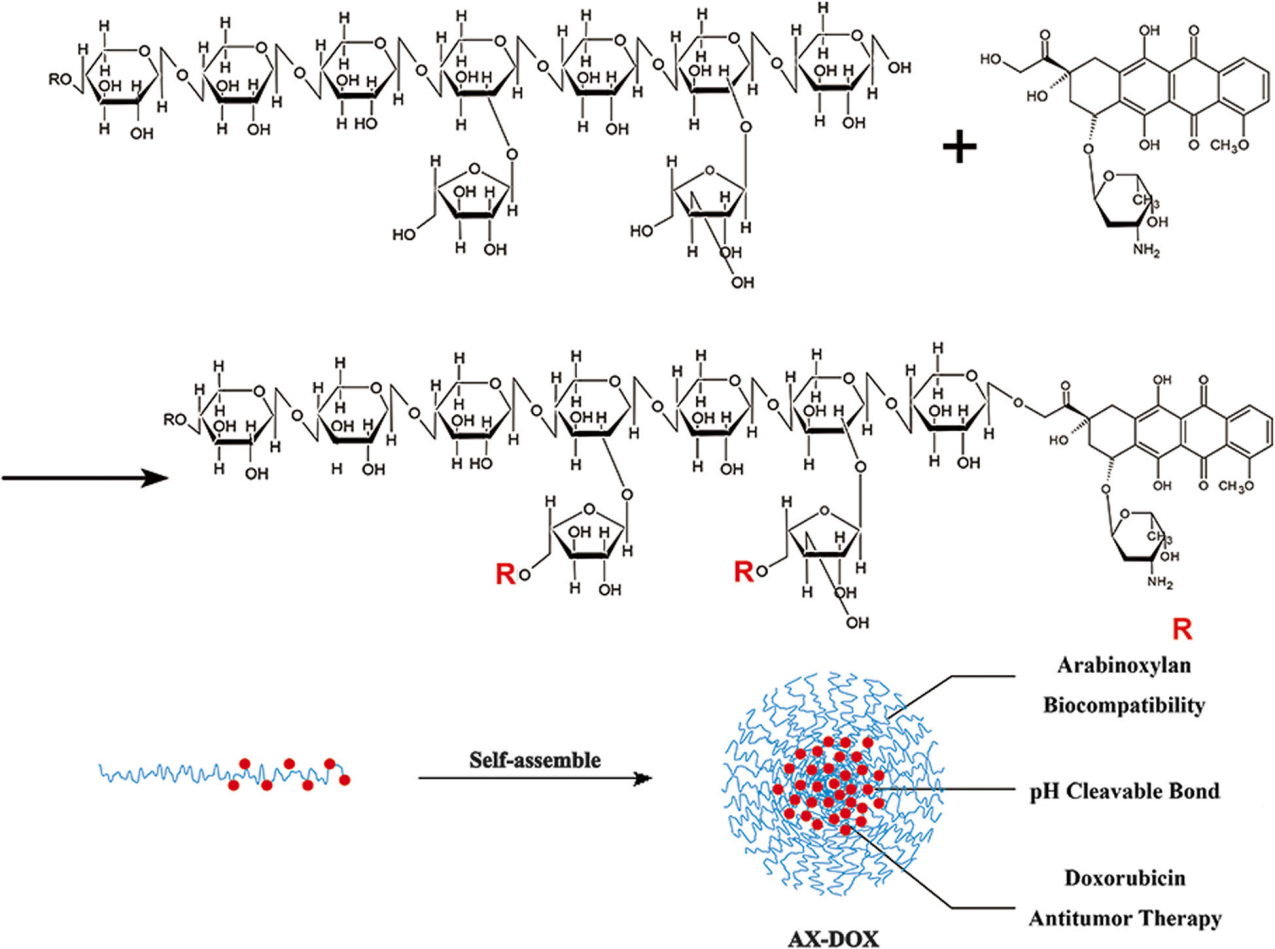
The scheme illustrated the synthesis and assembly process of micelles with well-defined structure

The synthesis of AX-drug conjugates was shown in Fig. 1. Shortly, the DOX·HCL was dehydrochlorinated by the TEA solution firstly. Then, the hydrophobic DOX was linked to the AX by the hydrogen bond. The well-defined core shell micelles were self-assembled from the amphiphilic AX-DOX chains by dialysis method. DOX was encapsulated inside of micelles through hydrogen bond and hydrophobic interaction. Noted here, the hydrogen bond is cleavable at low pH.

**Fig. 2:**
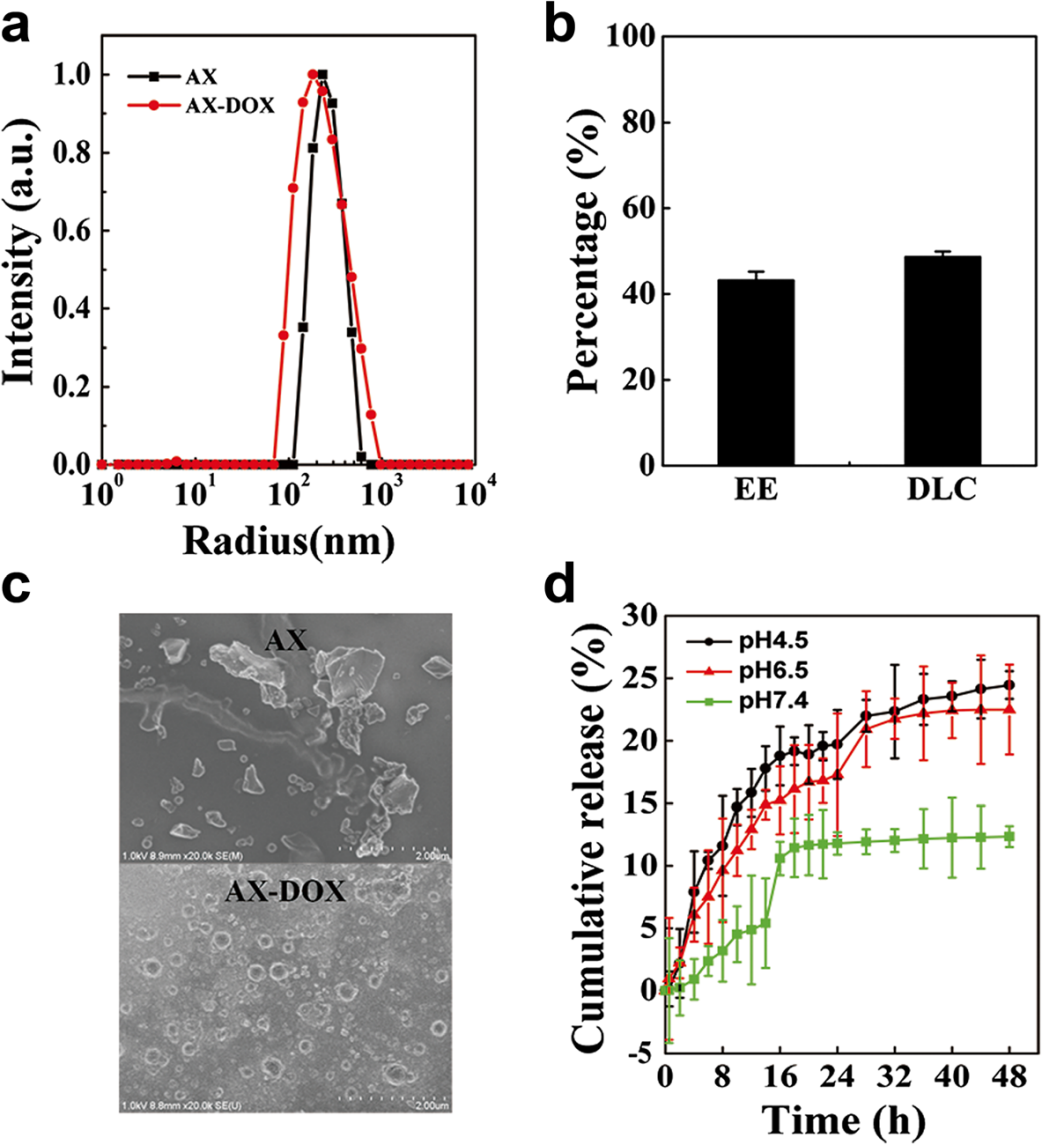
Characterization of micelles. **a** DLS testing of blank nanocarrier and DOX-loaded micelles in aqueous solution. **b** SEM images of AX and AX-DOX micelles showing their homogeneous size distribution. **c** The EE and DLC of DOX micelles. **d** The in vitro cumulative DOX released from the AX-DOX micelles in different pH media at RT over a time period of 48 h. Data are expressed as mean ± SD (*n* = 3)

Average hydrodynamic diameters, measured by dynamic light scattering (DLS), of blank nanocarrier and DOX-loaded micelles were all at the range from 20 to 1000 nm. Noted, we found the hydrodynamic diameter and size distribution of micelles after packaged DOX became smaller. These results suggested that the DOX-nanocarrier interactions through hydrogen bond and hydrophobic interaction can prevent the micelles from forming aggregates in aqueous solution and distributed more uniform [19] (Fig. 2a). Morphologically, scanning electron microscopy (SEM) images indicate that both the AX-DOX micelles have a narrow size distribution and AX have a anomalous formation (Fig. 2b). It is worth noting that the particle size of AX-DOX is more uniform than AX because the formation of hydrogen bond and hydrophobic interaction changes the original configuration of AX, prevents the aggregation, and promotes more uniform distribution. The EE and DLC of AX-DOX micelles are 43.12 ± 2.1% and 48.61 ± 1.3% (Fig. 2c). In addition, control experiments were performed at RT using 1 mg of DOX placed inside a dialysis bag, and the controlled drug release behavior from the DOX-loaded nanopatricles was investigated in three different buffer solutions (PBS/pH 7.4; PBS/pH 6.5; PBS/pH 4.5) individually. The cumulative release ratio of AX-DOX micelles was calculated to be approximately 12.3, 23.5, and 24.5% at pH 7.4, pH 6.5, and pH 4.5 within 48 h, respectively (Fig. 2d). Obviously, the micelles had a high release ratio in the acidic environment while a low DOX release in the neutral pH condition. That is, the release ratio increases with the decrease of the media pH value. The AX-DOX micelles are stability in neutral solution, limiting the release of DOX. However, the micelles can dissolve into the acidic media, which promotes hydrogen bonds cleavage [23], therefore the micelles are disintegrated and promote the release of DOX.

### In Vitro Cytotoxicity of the AX-DOX Micelles

**Fig. 3:**
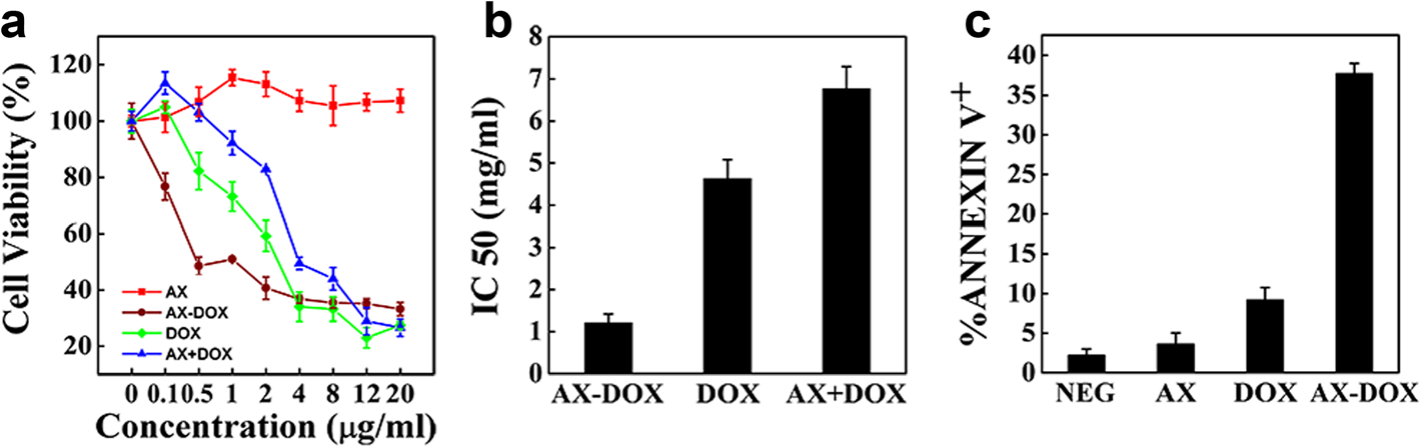
In vitro evaluation of the anti-proliferative efficacy of micelles. **a** The cytotoxic profile of blank nanocarriers, AX-DOX micelles, the DOX, and the blend AX with DOX at different concentrations after 48 h incubation with MCF-7. **b** IC_50_ value of the different DOX formulations. **c** The cell apoptosis induced by AX, DOX, and AX-DOX in MCF-7 cells; non-treated cells used as control. All values are presented as a mean SD (*n* = 3)

Different concentrations (0.1, 0.5, 1, 2, 4, 8, 12, 20 μg/mL) of the AX, DOX, AX-DOX, and the blend AX with DOX were prepared for evaluating biocompatibility of the AX and the cytotoxicity of AX-DOX against MCF-7 cells. These assays can help to distinguish whether blank nanocarrier is biocompatible and to evaluate the AX-DOX toxicity to tumor cells. Interestingly, it was observed that the cells treated with AX for 48 h were growth better (Fig. 3a) because of the fair nutritive value of AX. So, our nanocarrier has good biocompatibility and will not perform functions in loaded drugs’ chemotherapy. The effects of AX-DOX on proliferation inhibition of MCF-7 cells were also evaluated using CCK-8 assay after 48 h of treatment. As shown in Fig. 3a, AX-DOX micelles were much more effective at suppressing MCF-7 cells proliferation than other groups, especially when the concentration was lower than 4 μg/mL. As shown in Fig. 3b, the IC_50_ value of AX-DOX, DOX, and AX + DOX was 1.207, 4.633, and 6.776 μg/mL, respectively. Interestingly, the IC_50_ of AX + DOX was higher than other groups because of the fair nutritive value of AX. The IC_50_ in MCF-7 cells shown AX-DOX was 3.84-fold effective than DOX, indicating that the AX-DOX have much better antitumor therapeutic effect. The enhanced cytotoxicity of micelles over hydrophobic DOX is attributed to their enhanced cellular uptake, via slightly acidic environments, in the MCF-7 cells. The difference observed in cytotoxicity result from the different mechanism of cellular uptake for free drug versus the nanomedicine. The cellular uptake of free DOX occurs through a passive diffusion mechanism, while micelles are taken up by endocytosis, which overcome the low-efficiency problem. Therefore, these results demonstrate that micelles optimally inhibited MCF-7 cells proliferation in vitro.

The in vitro apoptosis-inducing capacity of AX-DOX was also evaluated via flow cytometry using 1 × 10^4^ MCF-7 cells for each sample. After staining, apoptotic cells were characterized based on Annexin V+ subsets. As shown in Fig. 3c, in comparison with the control group, after treatment with AX, DOX, and AX-DOX at concentration of 2 μg/mL for 48 h, the proportions of apoptotic cells were 2.16, 3.57, 9.16, and 37.64%, respectively. The increasing proportion of apoptotic cells induced by AX-DOX fully demonstrates that AX-DOX has a potent capacity in cancer therapy by proliferation inhibition and apoptosis.

### In Vitro Synergistic Cytotoxicity Induced by AX-DOX

**Fig. 4:**
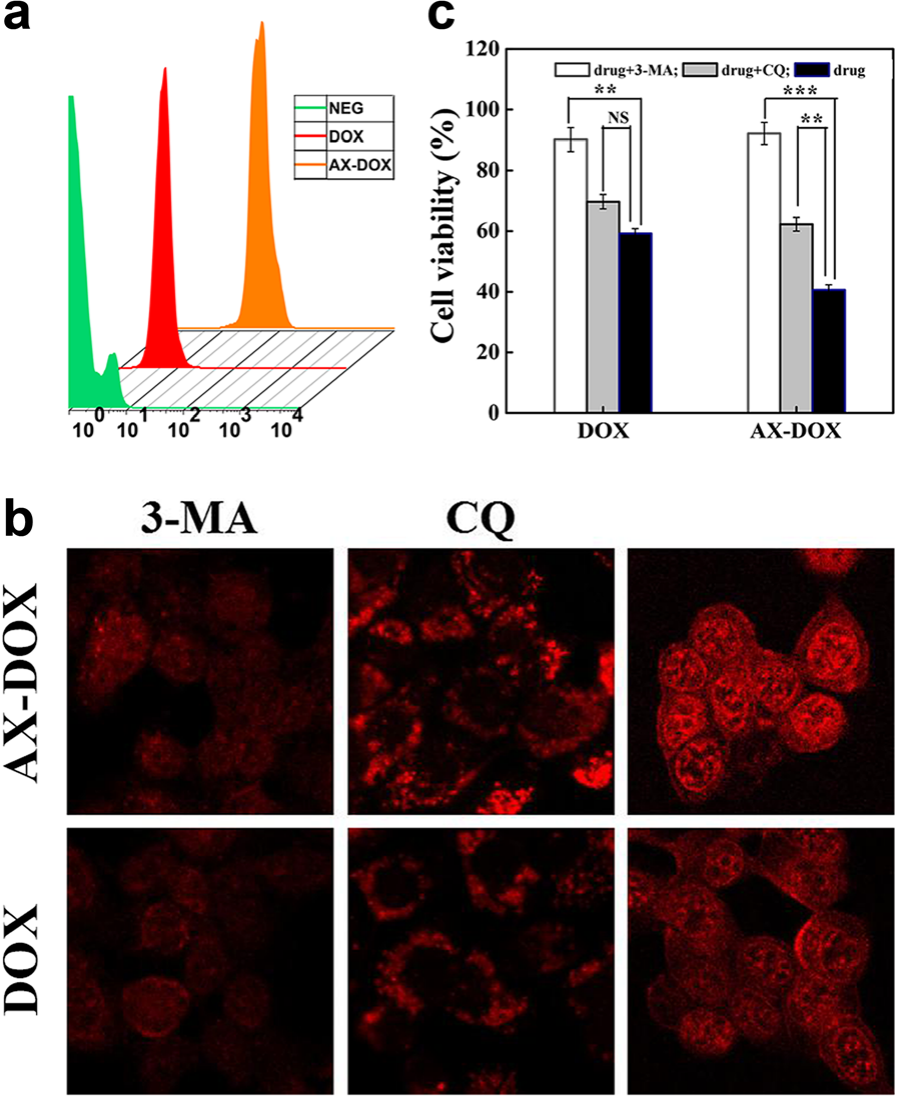
In vitro evaluation of the cellular uptake of micelles. **a** In vitro cellular uptake tested by flow cytometry for different DOX formulations. **b** CLSM images of MCF-7 cells. The cells were treated with AX-DOX, DOX at a concentration of 2 μg/mL and co-incubated with 10 mM 3-MA or 60 μM CQ for 8 h at 37 °C, respectively. **c** The in vitro cytotoxicity of the different DOX formulations under 10 mM 3-MA or 60 μM CQ to MCF-7 tumor cells. Data are expressed as mean ± SD (*n* = 3). *p < 0.05; **p < 0.01; ***p < 0.001; *NS* not significant

Based on the fluorescent intensity of the DOX, the cellular uptake of micelles was detected by flow cytometry and CLSM. The image shows that the cellular uptake of AX-DOX was higher than DOX (Fig. 4a, b). The CLSM images indicated that the release and distribution of micelles were different in diverse pH environment (Fig. 4b). 3-MA can suppress the autophagosomes formation, while CQ can block endosomes and autophagosomes fusion with lysosomes, therefore lead the significant accumulation of the autophagosomes [25]. It is well known that the pH value of the normal cells or tissues is ~7.3, while it is weakly acidic in tumor tissues (pH 6.2–6.9), especially in some organelles, such as endosomes (~pH 6.0), autophagosomes (~pH 5.0), and lysosomes (pH 4.5–5.0), the acidity is much higher [26]. Inhibition of autophagy can rescue the micelles from endosome and lysosome, and thus sustains the micelles existence in different organelles to evaluate the release of the drug under different pH. Our previous studies demonstrated that the size of micelles is small and the nanocarrier is hydrophilic with pH-sensitive to increase the cellular uptake of the micelles in MCF-7 cells. Moreover, the results (Fig. 4c) of cytotoxicity of the different DOX formulations under 3-MA or CQ further validate that the release ratio of drug increased with the decrease of the pH value. Thus, the cytotoxicity of AX-DOX was promoted by both the intratumor accumulation and the intracellular low pH.

### In Vivo Evaluation of the Antitumor Efficacy

**Fig. 5:**
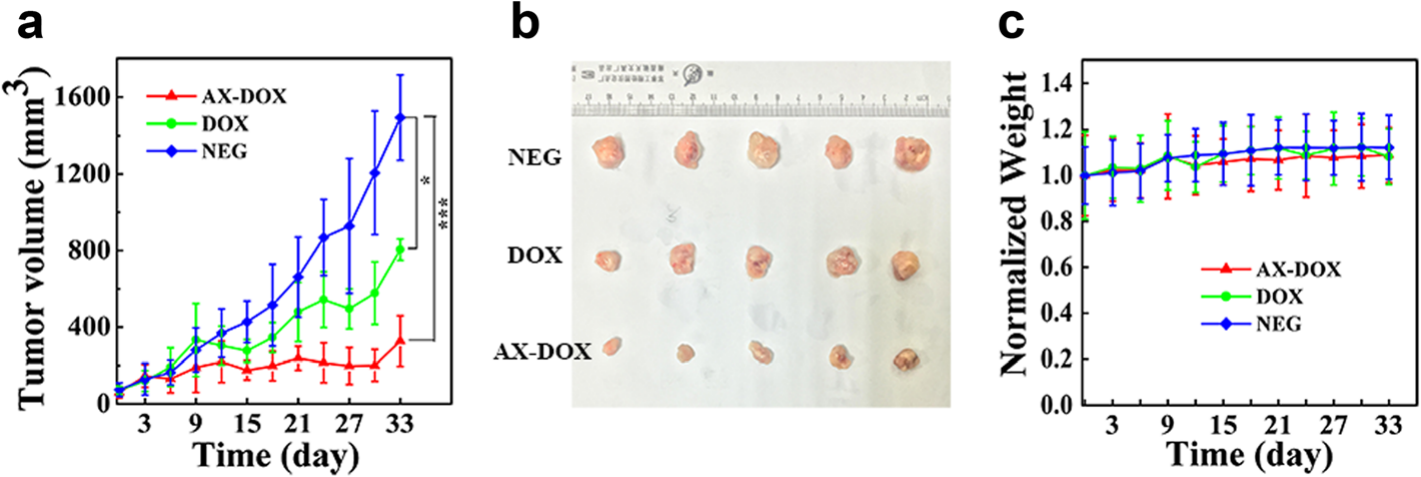
In vivo evaluation of the antitumor efficacy of micelles. **a** Tumor growth curves for the different DOX formulations with a dosage of 5 mg/kg and a total of three treatments. All values are presented as a mean SD (*n* = 5); *p < 0.05; **p < 0.01; ***p < 0.001; *NS* not significant. **b** Images of the tumors collected after sacrificing the mice on day 33. When tumors were established and reached ~50 mm^3^, mice were treated with the various drug formulations. The tumor volume (*V*) was calculated as: $V=\frac{\left( L\times {W}^2\right)}{2}$, where *L* is long diameter and *W* is short diameter of tumor determined using a caliper. **c** Change in the body weight of animals as a function of time

Our micelles were therefore applied to be administrated into Balb/c nude mice bearing MCF-7 cells to evaluate antitumor therapeutic effect. The change of tumor volume shows in Fig. 5a, b. All of the treatment groups exhibited inhibition of tumor growth as compared to the PBS control group. Overall, the AX-DOX micelles were the most effective at inhibiting tumor growth compared to the control, measured 33 days after the last injection. As expected, AX-DOX micelles induce much better antitumor efficacy than free DOX at the same dosage, which is consistent with the in vitro CCK-8 assay findings. This observation can be largely contributed to the fact that the AX-DOX can significantly enhance the tumor cellular accumulation. In other words, the cellular uptake of the micelles in MCF-7 cells is dramatically enhanced compared to that of free DOX, which is partly attributed to the enhanced permeability and retention (EPR). In addition, as the micelles enter into cells, it will encounter a low-pH environment. This is because the weakly acid environment in tumor tissues (pH 6.2–6.9), especially in some organelles, such as endosomes and lysosomes, the acidity is much higher (pH 4.5–6.0). The acidic environment promotes the hydrogen bond breaking, which results in the degradation of micelles and successful intracellular release drug. Furthermore, during the whole treatment process, there were not any noticeable changes in body weight (Fig. 5c), indicating that the AX-DOX system is safe.

**Fig. 6:**
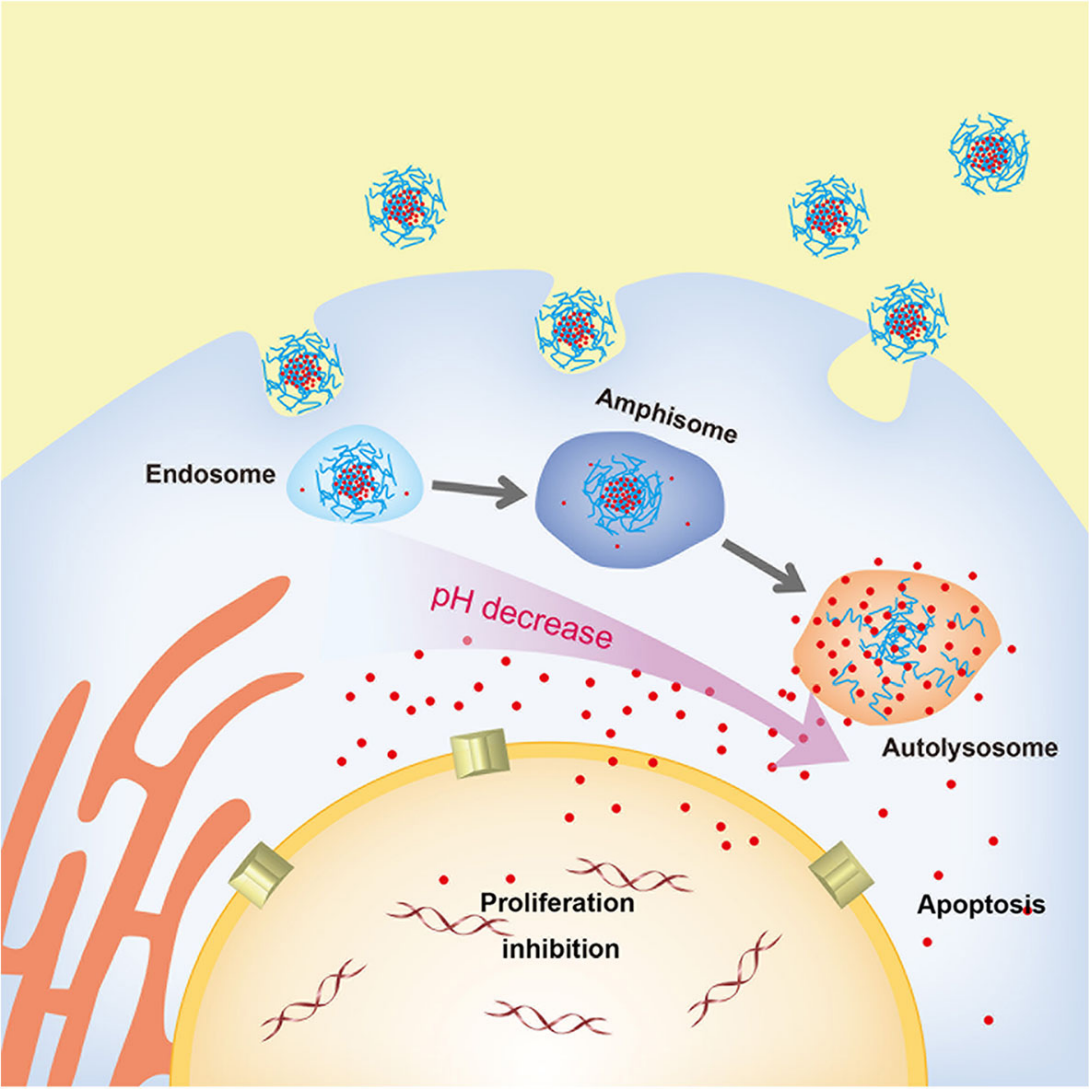
Scheme illustrated the cellular level mechanism of the AX-DOX formulation

Consequently, the intratumor accumulation of AX-DOX is attributed to the well-known EPR. Meanwhile, the AX-DOX with pH-cleavable bond can promote the release of the drug in tumor accompanied with obvious apoptosis (Fig. 3c). As schemed in Fig. 6, the therapeutic effects of AX-DOX are attributed to both the proliferation inhibition and apoptosis, which was further synergistically promoted by the EPR and intracellular pH-triggered drug release. All the results indicate that such AX-DOX micellular formulation held high potential in cancer therapy.

## Conclusions

In this work, by employing AX as a natural nanocarrier encapsulating DOX, the AX-DOX micelles with pH-sensitivity, and high biocompatibility were synthesized and characterized for MCF-7 breast cancer synergistic therapy. The DOX release from AX-DOX micelles is dependent upon the cleavage of hydrogen bonds and drug-nanocarrier interactions, which are influenced by the environmental pH. The in vitro cytotoxicity against MCF-7 cells showed that such AX-DOX micelles dramatically enhanced the cellular uptake of DOX, owing to the synergistic effects of proliferation inhibition and apoptosis. Consequently, the in vivo tumor inhibition by this AX-DOX was dramatically promoted, indicating that the AX-DOX exhibited a significantly higher tumor accumulation and better antitumor efficacy than DOX. These in vitro/vivo results indicated that the improved therapeutic effect of AX-DOX over DOX in MCF-7 cells is attributed to the good pH-triggered drug release capability, excellent biocompatibility, and effective antitumor activity of the AX-DOX micelles.

## Abbreviations

3-MA: 3-Methyladenine
AX: Arabinoxylan
CCK-8: Cell counting kit-8
CLSM: Confocal laser scanning microscope
CQ: Chloroquine
DLC: Drug loading capacity
DLS: Dynamic light scattering
DMAC: *N*,*N*-dimethylacetamide
DMEM: Dulbecco’s modified Eagle’s medium
DOX: Doxorubicin
DOX∙HCl: Doxorubicin hydrochloride
EE: Encapsulation efficiency
EPR: Enhanced permeability and retention
FBS: Fetal bovine serum
MCF-7: Breast cancer cells
MWCO: Molecular weight cutoff
RT: Room temperature
SD: Standard deviation
SEM: Scanning electron microscopy
SPF: Specific pathogen-free
TEA: Triethanolamine
